# Molecular interactions of STAC proteins with skeletal muscle dihydropyridine receptor and excitation‐contraction coupling

**DOI:** 10.1002/pro.4311

**Published:** 2022-04-20

**Authors:** Dmitry Shishmarev, Emily Rowland, Shouvik Aditya, Srinivasan Sundararaj, Aaron J. Oakley, Angela F. Dulhunty, Marco G. Casarotto

**Affiliations:** ^1^ John Curtin School of Medical Research Australian National University Canberra Australia; ^2^ Research School of Biology Australian National University Canberra Australia; ^3^ Division of Structural Biology, Wellcome Centre for Human Genetics University of Oxford Oxford UK; ^4^ School of Chemistry & Molecular Bioscience and Molecular Horizons University of Wollongong Wollongong Australia

**Keywords:** DHPR, excitation‐contraction coupling, II–III loop, protein–protein interactions, skeletal muscle, STAC2, STAC3

## Abstract

Excitation‐contraction coupling (ECC) is the physiological process in which an electrical signal originating from the central nervous system is converted into muscle contraction. In skeletal muscle tissue, the key step in the molecular mechanism of ECC initiated by the muscle action potential is the cooperation between two Ca^2+^ channels, dihydropyridine receptor (DHPR; voltage‐dependent L‐type calcium channel) and ryanodine receptor 1 (RyR1). These two channels were originally postulated to communicate with each other via direct mechanical interactions; however, the molecular details of this cooperation have remained ambiguous. Recently, it has been proposed that one or more supporting proteins are in fact required for communication of DHPR with RyR1 during the ECC process. One such protein that is increasingly believed to play a role in this interaction is the SH3 and cysteine‐rich domain‐containing protein 3 (STAC3), which has been proposed to bind a cytosolic portion of the DHPR α_1S_ subunit known as the II–III loop. In this work, we present direct evidence for an interaction between a small peptide sequence of the II–III loop and several residues within the SH3 domains of STAC3 as well as the neuronal isoform STAC2. Differences in this interaction between STAC3 and STAC2 suggest that STAC3 possesses distinct biophysical features that are potentially important for its physiological interactions with the II–III loop. Therefore, this work demonstrates an isoform‐specific interaction between STAC3 and the II–III loop of DHPR and provides novel insights into a putative molecular mechanism behind this association in the skeletal muscle ECC process.

**Abbreviations**
CSP: chemical shift perturbation
DHPR: dihydropyridine receptor
DTT: dithiothreitol
DSS: sodium trimethylsilylpropanesulfonate
ECC: excitation‐contraction coupling
EDTA: ethylenediaminetetraacetic acid
HSQC: heteronuclear single‐quantum coherence
IPTG: isopropyl β‐d‐1‐thiogalactopyranoside
NAM: Native American myopathy
NMR: nuclear magnetic resonance
PKC: protein kinase C
RyR1: ryanodine receptor 1
SDS‐PAGE: sodium dodecyl sulphate‐polyacrylamide gel electrophoresis
SH3: Src homology three
SPR: surface plasmon resonance
STAC: SH3 and cysteine‐rich domain

## 1 INTRODUCTION

SH3 and cysteine‐rich domain‐containing (STAC) proteins form a small group of adaptor proteins comprised of STAC1, STAC2, and STAC3.^1^, ^2^ While STAC1 and STAC2 are predominantly found in neuronal tissue, STAC3 is exclusively expressed in skeletal muscle.^3^ In recent years, STAC3 has been touted as a potential “missing link” in skeletal muscle excitation‐contraction coupling (ECC), where it has been proposed to mediate the interactions between the voltage‐sensing dihydropyridine receptor (DHPR) and Ca^2+^‐releasing ryanodine receptor 1 (RyR1).^4^, ^5^, ^6^, ^7^ The first member of the STAC family of proteins (STAC1) was discovered in 1996 using a genetic screen of mouse neural tissue,^8^ while STAC2 and STAC3 were subsequently identified in 2010 in a targeted search for proteins demonstrating considerable sequence homology to STAC1.^9^ STAC3 knockouts in various animal models (e.g., mice, salmon, and zebrafish) were shown to cause impaired muscle development, musculoskeletal defects, and diminished ECC capacity.^3^, ^10^, ^11^ Moreover, point mutations in human STAC3 have been linked to myopathies, for example, the W284S mutation has been shown to be the cause of Native American myopathy (NAM).^12^, ^13^

The studies of Horstick et al.^11^ and Nelson et al.^3^ were among the first to characterize STAC3, suggesting that it is primarily localized at the transverse tubules of skeletal muscle tissue. Knockout of STAC3 in mice diaphragm muscles led to impaired ECC, with no muscle contractions observed following electrical stimulation, despite normal neuromuscular junctions, neurotransmission, and contraction upon application of a RyR1 agonist.^3^ In 2015, Polster and co‐workers demonstrated that STAC proteins were involved in the trafficking of DHPR to the plasma membrane of tsA201 human embryonal kidney cells, and DHPR localized to the endoplasmic reticulum in cells that lacked STAC3 expression.^14^ As a result, cells expressing both DHPR and STAC3 exhibited robust Ca^2+^ currents that were absent in cells without STAC3, highlighting the role of STAC3 in supporting functional membrane expression of DHPR.^14^ Similar to the experiments in tsA201 cells, knockout of STAC3 in myotubes resulted in diminished surface expression of DHPR and reduced Ca^2+^ currents.^4^ However, the reduction in Ca^2+^ transients was greater than would be expected if it was caused solely by the decreased DHPR membrane expression, suggesting that STAC3 played an additional direct role in mediating Ca^2+^ release and the ECC process in general.^4^ Like STAC3, STAC1 and STAC2 were also shown to support membrane expression of DHPR and facilitate depolarization‐evoked Ca^2+^ release.^5^

**FIGURE 1:**
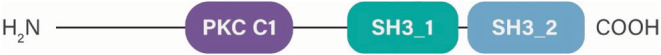
Structural domains of STAC proteins. The three STAC proteins share common structural features including a protein kinase C‐like C1 domain (PKC C1) and two SH3 domains (SH3_1 and SH3_2) at the C‐terminal end, collectively referred to as the tandem SH3 domains (STACSH3s)

Structurally, STAC proteins are composed of a PKC C1 (zinc finger) domain, flanked by intrinsically disordered regions and tandem SH3 domains at the C‐terminal end (Figure 1).^3^, ^6^, ^8^ In 2017, Wong King Yuen and co‐workers determined the X‐ray crystal structures of tandem‐SH3 domains of STAC1 and STAC2 and the structure of the individual second SH3 domain (SH3_2) of STAC3.^6^ Additionally, the X‐ray crystal structure of STAC2 in complex with a peptide from the cytosolic II–III loop of the main pore‐forming subunit of DHPR (α_1S_) suggested that an interaction between STAC2 and DHPR was mostly mediated by the first SH3 (SH3_1) domain of STAC2.^6^ These structural studies were consistent with isothermal titration calorimetry experiments demonstrating low micromolar binding of all three STAC proteins to residues 728–775 of the II–III loop.^6^ The shortest peptide of the II–III loop that maintained an ability to bind the STAC proteins consisted of residues 747–760 (EDEPEIPLSPRPRP), and this peptide bound to the isolated first SH3 domain of STAC2 (STAC2 SH3_1), but not SH3_2. Two proline residues in this peptide sequence of the II–III loop (P756 and P758) were shown to be essential for the interaction with STACs. In fact, all three STAC isoforms were demonstrated to interact with the short peptide sequence of the DHPR II–III loop containing these two crucial proline residues.^6^ However, STAC3 was shown as the only STAC isoform that associated with DHPR in dysgenic myotubes, with STAC1 and STAC2 showing little or no association, and thus being unable to reconstitute the EC coupling.^15^

In 2018, Polster and co‐workers studied chimeras between skeletal and cardiac variants of DHPR II–III loop proteins and found that only constructs containing residues 720–765 of the skeletal DHPR (α_1S_) II–III loop were able to associate with STAC3 and facilitate ECC.^5^ The authors suggested that residues 745–765 are the most critical for STAC binding, in agreement with the findings of Wong King Yuen et al.^6^ Furthermore, Polster and co‐workers confirmed that the interaction of STAC3 with the II–III loop requires the SH3_1 domain.^5^ They described an interaction between the first SH3 domain of STAC proteins and a short peptide sequence of the II–III loop, which had been previously postulated to mediate ECC in skeletal muscle.^16^ Indeed, this interaction between STAC3 and the II–III loop was shown to be important for facilitating ECC in cultured myotubes.^5^ Interestingly, the STAC PKC C1 domain also plays an important role in forming a stable association with another region of DHPR.^15^ This interaction is mediated by the C‐terminal domain of DHPR and modulates the channel activity by inhibiting Ca^2+^‐dependent inactivation, but it does not directly participate in the skeletal muscle ECC.^17^

While the importance of STAC3 in skeletal muscle physiology is now well established, the biological relevance of the neuronal isoforms of STAC remains ambiguous, with implications including osteoclast formation, promotion of Ca^2+^ channel surface expression, heart failure, and nerve injury.^18^, ^19^, ^20^, ^21^ Despite structural similarities between the three STAC isoforms,^6^ their functional differences are at times striking and the subject of several investigations to date.^5^, ^6^, ^14^, ^22^ Potentially, such contrasts can provide valuable information into the functional role of STAC3 in the context of skeletal muscle ECC. Notably, even though STAC1 and STAC2 SH3 domains are readily crystallized, the wild‐type STAC3 tandem‐SH3 domains have proved resistant to crystallization,^6^ and only a mutant of STAC3SH3s (P269R) has been recently crystallized.^23^ These crystal structures of STAC proteins enabled conjecture on their functional features and led to the hypotheses accounting for the molecular mechanism of their interaction with the DHPR.^6^, ^23^ Nevertheless, these structures do not take into account the dynamic nature and behavior of STAC proteins under native solution‐state conditions, and specific residues of STAC3 and the II–III loop that determine their interaction during the skeletal ECC are yet to be fully elucidated.

The aim of the present work was to illuminate the molecular and structural mechanisms of II–III loop binding to the STAC SH3 domains using Surface Plasmon Resonance (SPR), Nuclear Magnetic Resonance (NMR) spectroscopy, and modeling using AlphaFold.^24^ As a result, we have identified the specific amino acid residues in STAC2 and STAC3 isoforms and the DHPR II–III loop mediating their interaction in the solution state.

## 2 RESULTS

### 2.1 STAC2 and STAC3 isoforms bind DHPR II–III loop with comparable affinity

**FIGURE 2:**
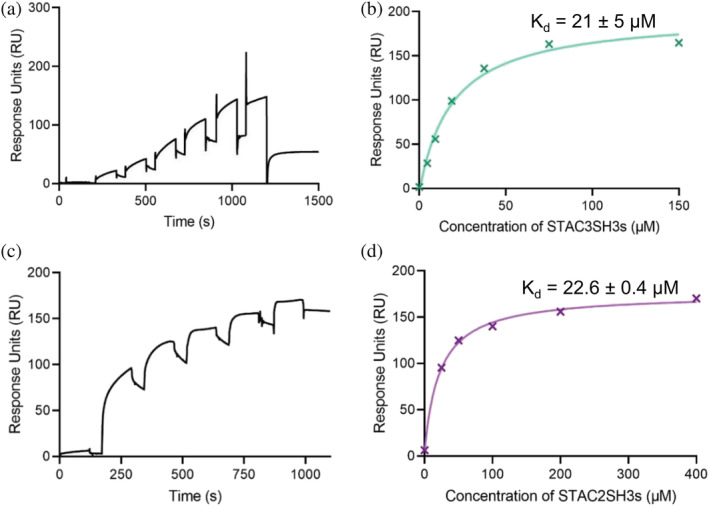
Tandem SH3 domains of STAC2 and STAC3 bind DHPR II–III loop peptide with a micromolar affinity. Binding of the full‐length DHPR II–III loop to STAC proteins was established using SPR spectroscopy. The II–III loop was immobilized via amine coupling to a CM5 sensor chip, and increasing concentrations of STAC proteins in HEPES‐buffered saline were applied. (a) Typical sensorgram of immobilized II–III loop titrated with STAC3SH3s. (b) Affinity curve of STAC3SH3s binding to the II–III loop, derived from A, which indicates a binding affinity of 21 ± 5 μM (*n* = 3). (c) Representative sensorgram of II–III loop titrated with STAC2SH3s. (d) Typical affinity curve for STAC2SH3s fitted to the data points derived from the sensorgram in C. The determined affinity of binding between STAC2SH3s and the II–III loop was 22.6 ± 0.4 μM (*n* = 2)

Based on the previous suggestions that the tandem SH3 domains of all three STAC isoforms interact with the II–III loop of DHPR,^6^ we first endeavored to confirm these interactions using SPR spectroscopy. Increasing concentrations of STAC isoforms were passed over the immobilized full‐length II–III loop (115 amino acids; see Figure S1), which was bound to a CM5 sensor chip via amine coupling. As shown in Figures 2a, b, STAC3SH3s bound to the II–III loop with an affinity K_d_ of 21 ± 5 μM, while STAC2SH3s bound to the II–III loop with a comparable affinity of 22.6 ± 0.4 μM; Figure 2c, d).

### 2.2 STAC3SH3s bind to a stretch of consecutive amino acids in II–III loop

**FIGURE 3:**
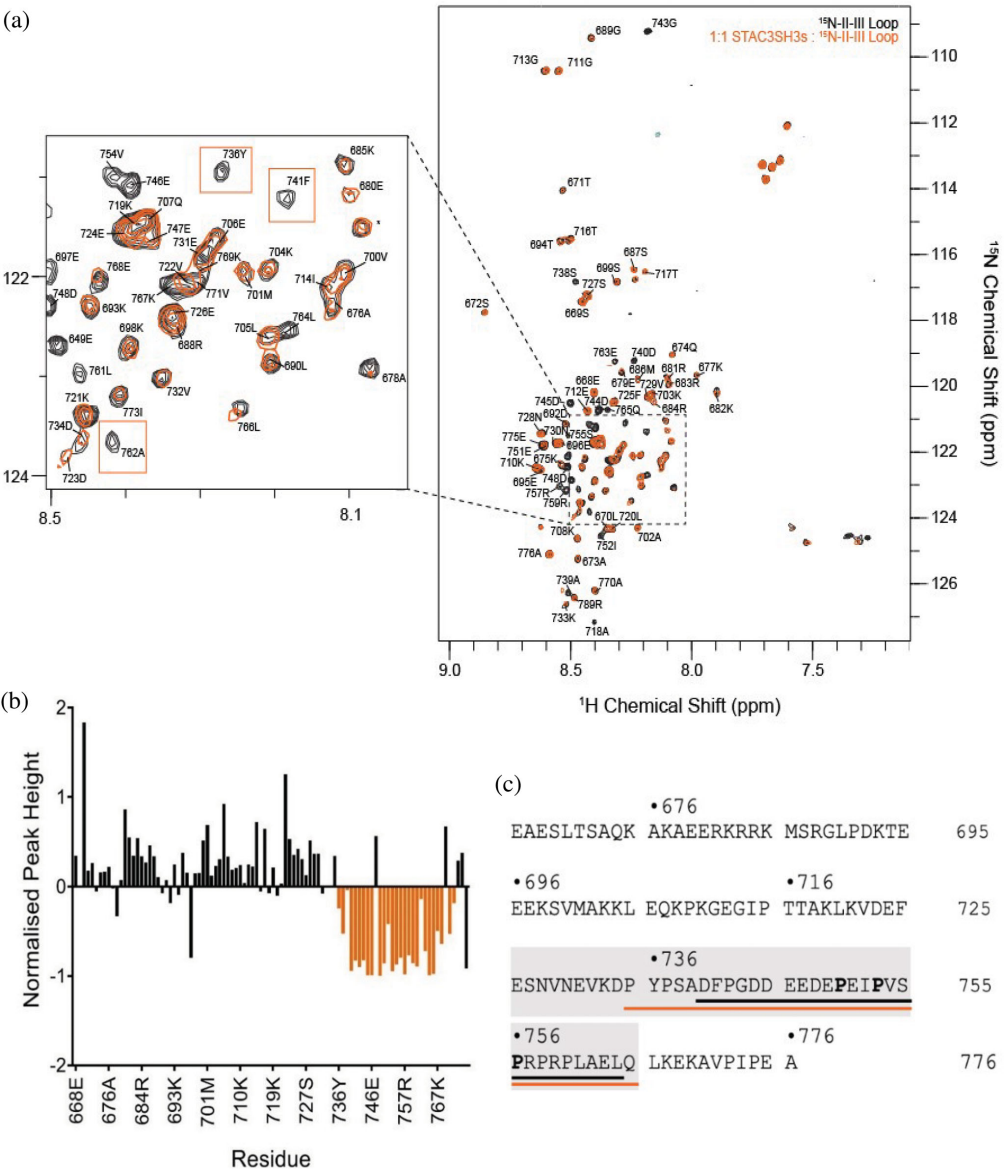
A ~ 30 amino acid sequence of II–III loop directly interacts with STAC3SH3s. (a) ^1^H‐^15^N HSQC NMR spectra of 0.17 mM ^15^N‐labeled II–III loop in the presence (orange) and absence (black) of STAC3SH3s (in 1:1 ratio), in 27 mM NaH_2_PO_4_, 30 mM Na_2_HPO_4_, 200 mM KCl, 10% D_2_O, 0.1 mM DSS buffer (pH 6.5). Examples of peaks that disappear upon the addition of STAC3SH3s are highlighted by orange boxes. Peaks are annotated with sequence‐specific assignments from Cui et al.^33^ with their numbers adjusted to refer to the position in the sequence of full α_1S_‐subunit of DHPR. (b) Peak heights in NMR spectra of DHPR II–III loop with STAC3SH3s (orange spectrum in A), normalized to the respective heights of peaks in the reference spectrum of the II–III loop (black spectrum in A). Several consecutive amino acid residues with reduced peak intensities upon binding of STAC3SH3s are highlighted in orange. (c) Amino acid sequence of the full‐length DHPR II–III loop. The region purported to interact with STAC3SH3s is highlighted by the orange line. The amino acid sequence of the peptide used in the following experiments is highlighted by the black line. The amino acid sequence of the peptide used in the work of Wong King Yuen et al.^6^ is highlighted by the grey box. Three proline residues in PXXP motifs (canonical SH3 binding motifs) are bolded

Following estimation of the binding affinities between STAC proteins and the full II–III loop, ^1^H‐^15^N HSQC NMR spectroscopy was pursued to identify specific amino acid residues within the II–III loop that are directly involved in the interaction with STAC3SH3s in the solution state. The full‐length DHPR II–III loop construct was titrated with STAC3SH3s up to a 1:1 ratio, which led to significant reductions in the intensity of peaks corresponding to a consecutive stretch of amino acid residues in the sequence of the II–III loop. Figure 3a illustrates the HSQC NMR spectra of the ^15^N‐labeled II–III loop in the presence (orange) and the absence (black) of STAC3SH3s (1:1 ratio), which shows the reduction in the intensity or complete disappearance of several NMR peaks, signifying involvement of these residues in the interactions.

The measured reduction in the peak intensity plotted against the amino acid sequence of the DHPR II–III loop is shown in Figure 3b. The region of the sequence containing a notable cluster of reduction in peak intensity is highlighted in orange on the full amino acid sequence of the DHPR II–III loop in Figure 3c (residues 735–765). This region contains two canonical PXXP motifs known to be involved in SH3 domain protein–protein interactions.^25^ This amino acid region is contained within the II–III loop peptide (residues 720–765), identified as critical for skeletal ECC by Grabner et al. in 1999,^16^ and it corresponds well to the “minimal peptide” sequence used in Wong King Yuen et al.^6^ (residues 747–760) to demonstrate binding of the II–III loop to the STAC proteins. Additionally, it matches well with a small peptide known as the “C3 peptide” (residues 740–764), which was chosen to be used in subsequent NMR titration experiments in this work (Figure 3c).

### 2.3 Isoform‐specific interactions of STAC3SH3s with the II–III loop C3 peptide

**FIGURE 4:**
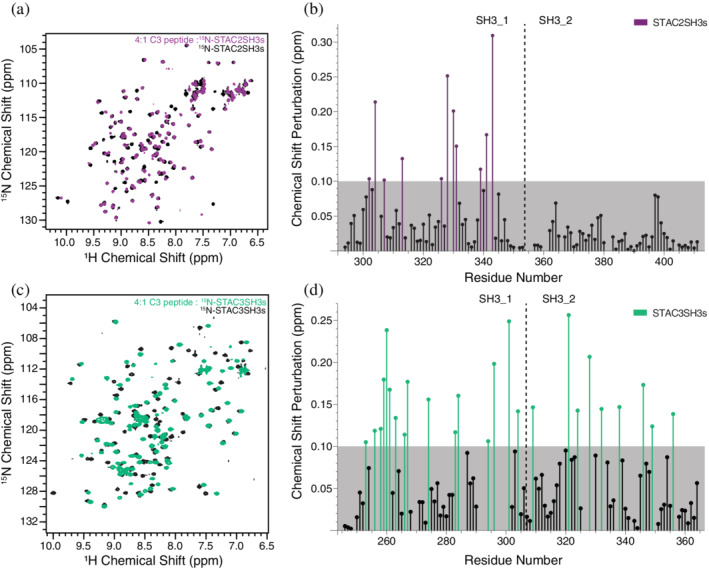
Interactions of STAC2SH3s and STAC3SH3s with II–III loop C3 peptide. (a) ^1^H‐^15^N HSQC NMR spectra of 0.2 mM ^15^N‐STAC2SH3s in the presence (purple) and absence (black) of the C3 peptide (final 4:1 ratio of C3:STAC2SH3s). (b) CSPs measured from the spectra in (a), plotted against the amino acid sequence of STAC2SH3s. The grey box delineates the cutoff of 0.1 ppm, and the purple bars indicate significant CSPs that exceed this threshold. (c) ^1^H‐^15^N HSQC NMR spectra of 0.2 mM ^15^N‐STAC3SH3s in the presence (green) and absence (black) of the C3 peptide (final 4:1 ratio of C3:STAC3SH3s). (d) CSPs measured from the spectra in (c), plotted against the amino acid sequence of STAC3SH3s. The grey box delineates the cutoff of 0.1 ppm, and the green bars indicate significant CSPs that exceed this threshold

Following the identification of a region within the II–III loop that directly interacts with STAC3SH3s, we attempted to clarify the corresponding binding regions within STAC proteins and the mode of this interaction. ^15^N‐labeled STAC2SH3s and STAC3SH3s were titrated with increasing concentrations of the II–III loop C3 peptide (1:0.5, 1:1, 1:2, 1:4 STAC:peptide ratios). NMR chemical shift perturbations were observed in the spectra of both STAC2SH3s (Figure 4a) and STAC3SH3s (Figure 4c), albeit with differences in the number and distribution of chemical shift perturbations (CSPs). STAC3SH3s displayed a greater number of CSPs (Figure 4d) than STAC2SH3s, which presented with CSPs within only the first SH3 domain (Figure 4b), in contrast to those of STAC3SH3s, which spanned both SH3 domains (Figure 4d).

**FIGURE 5:**
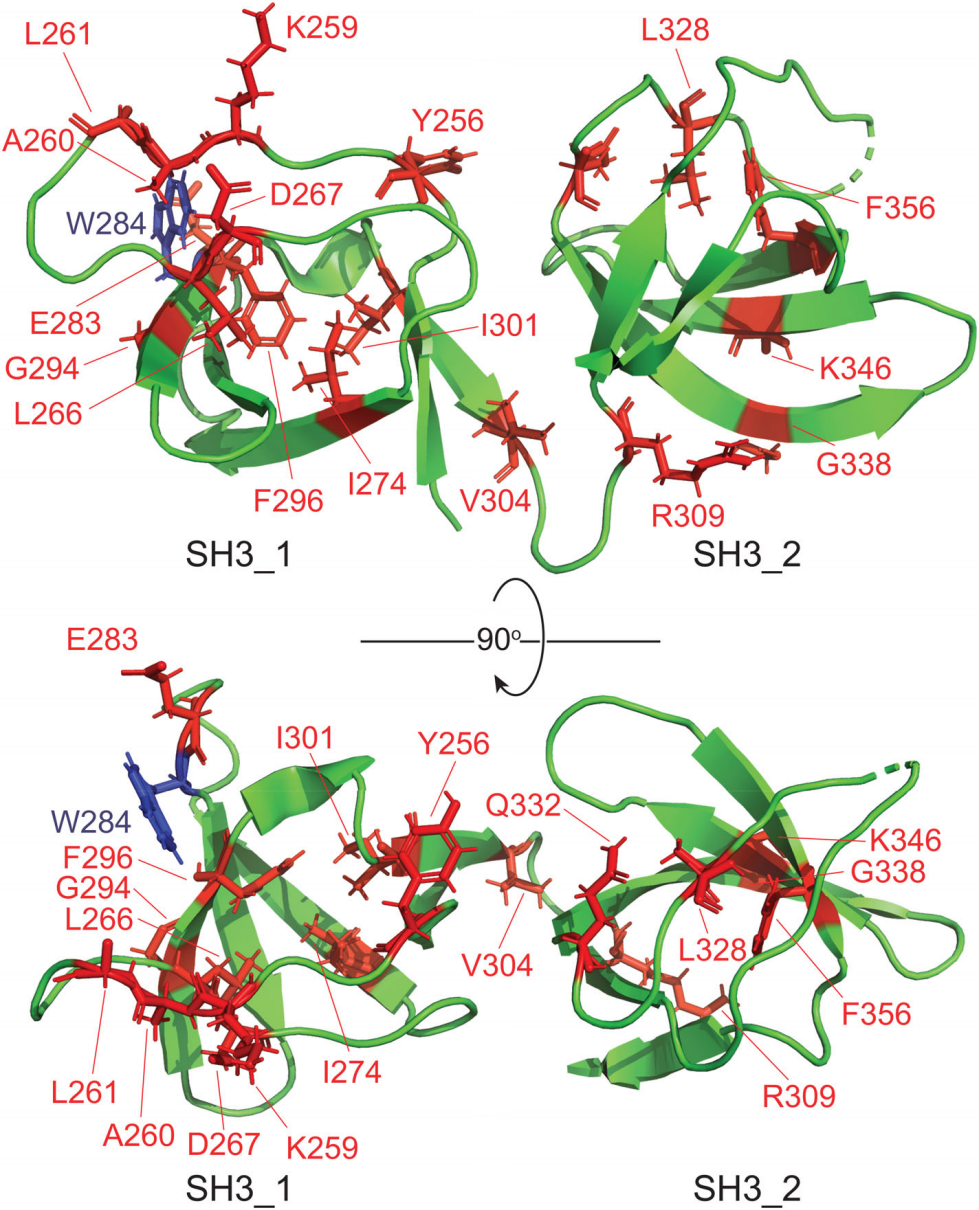
Amino acid residues of STAC3SH3s involved in the interaction with the C3 peptide. Amino acid residues of STAC3SH3s that exhibited a significant CSP in the NMR titration experiment with the C3 peptide (Figure 4d) are highlighted in red color and have their side chains shown as “sticks”. The crystal structure of the STAC3SH3s P269R mutant (PDB: 6UY7)^23^ was used for residue mapping. W284 residue, which is mutated in NAM, is highlighted in blue. The figure was prepared using the software PyMOL 2.4

The amino acid residues of STAC3SH3s that displayed significant CSPs were mapped on the crystal structure of the STAC3SH3s P269R mutant (PDB: 6UY7)^23^ (Figure 5). Mapping revealed a notable cluster of interacting residues in the vicinity of the residue W284 (canonical mutation in NAM) in the SH3_1 domain of STAC3. This implies that the physiological binding of the II–III loop to STAC3SH3s might be compromised in NAM due to the mutation of W284 or other residues in its vicinity. Nevertheless, the amino acid residues of STAC3SH3s displaying significant CSPs were scattered across the two SH3 domains, suggesting that both SH3 domains of STAC3 are involved in interaction with DHPR in a tandem.

Following these results, we also attempted to study interactions of individual SH3 domains of STAC3 with the C3 peptide. While we were unable to express the individual SH3_1 domain of STAC3 due to its instability, titration of the solitary SH3_2 domain of STAC3 with the C3 peptide of the II–III loop resulted in no significant CSPs (see Figure S2), suggesting that SH3_2 alone is not sufficient, and SH3_1 is required for interactions of STAC3SH3s with the II–III loop.

### 2.4 AlphaFold modeling predicts interactions between peptide C3 and both SH3 domains of STAC3

To further elucidate differences in the interactions of the DHPR II–III loop with both STAC2 and STAC3, we employed modeling using AlphaFold‐multimer.^26^ This modeling predicted interactions between the peptide C3 region of the II–III loop and both SH3 domains of STAC3 (Figure S3A). In contrast, when STAC2 was used in the modeling, the C3 peptide was predicted to interact with the first SH3 domain only (Figure S3B). These results are consistent with the NMR titration data, which revealed CSPs for the SH3_1 domain only for STAC2, and CSPs for both SH3_1 and SH3_2 in the case of STAC3.

## 3 DISCUSSION

ECC translates an electrical signal from the central nervous system into the contraction of skeletal muscles via mechanical interactions between two Ca^2+^ channels, DHPR, and RyR1. These two channels have been proposed to be physically coupled via the adaptor protein STAC3. Recent reports have suggested that the first SH3 domain of STAC3 binds to a cytoplasmic loop of DHPR to facilitate ECC. However, the exact nature of the interactions between STAC3 and these two Ca^2+^ channels has remained ambiguous. In the present work, we show the interaction of STAC3 with the II–III loop of DHPR to be mediated by a small peptide in the C‐terminal region of the II–III loop (C3 peptide). In contrast to previous studies, we reveal that this interaction involves the participation of both SH3 domains of STAC3, potentially involving a global conformational change in the tandem‐SH3 domains of STAC3 upon binding of the II–III loop.

### 3.1 STAC isoforms bind DHPR II–III loop

To characterize the interaction of the tandem‐SH3 domains of STAC proteins with the II–III loop of DHPR, we performed SPR affinity measurements, which revealed micromolar binding for STAC2SH3s and STAC3SH3s with a comparable affinity (Figure 2), despite the fact that STAC2 is not a native binding partner of the skeletal DHPR II–III loop. While reaffirming previous findings that STAC proteins interact with the II–III loop, the K_d_ values in the present work differ from those of Wong King Yuen et al.^6^ This discrepancy is likely due to the fact that we used the full‐length II–III loop, while Wong King Yuen et al.^6^ used a 47‐residue construct of the II–III loop, which is <50% of the full construct. Nevertheless, our results confirmed an association between the II–III loop and the STAC proteins, an interaction that is, in the case of STAC3, likely to be important for skeletal muscle ECC.^4^

### 3.2 STAC3SH3s bind to a small sequence of consecutive amino acids of the DHPR II–III loop

Direct interactions of STAC3SH3s with the full‐length DHPR II–III loop in solution were confirmed by using NMR titration experiments with ^15^N‐labeled II–III loop and STAC3SH3s. Our previous NMR structural study indicated the presence of a loosely globular structure of the full II–III loop construct.^27^ Significantly, present results show that only a sequence of ~30 amino acid residues in the C‐terminal region of the II–III loop (C3 peptide) is directly involved in binding STAC3SH3s, and no other segments of the full II–III loop participate in this interaction. This interacting region is consistent with those proposed by Grabner et al.,^16^ Wong King Yuen et al.,^6^ and Polster et al.^5^ as being crucial for binding to STAC3.

Furthermore, the location of the binding sequence (C3 peptide) at the C‐terminus of the II–III loop potentially makes geometric sense in the context of the work demonstrating stable binding of the STAC3 PKC C1 domain to the C‐terminal domain of voltage‐gated Ca^2+^ channels, which is a region located downstream of the II–III loop.^17^, ^28^ That is, initial binding of STAC3 to the C‐terminal tail of DHPR would situate the adaptor protein closer to the C‐terminus of the II–III loop, thus enabling this interaction during the ECC process.

Thus, a major finding of this study is that SH3 domains of STAC3 interact exclusively with the ~30 amino acid peptide of the II–III loop C‐terminus (residues 735–765), with no evidence of other residue participation outside this region. This peptide sequence of the DHPR II–III loop (C3 peptide) was used in subsequent titration experiments to elucidate the regions in STAC proteins, important for this interaction.

### 3.3 Differences in the extent of binding of the II–III loop with STAC2SH3s and STAC3SH3s

The C3 peptide was used to investigate corresponding binding regions within the STAC2 and STAC3 isoforms. NMR spectroscopy titration experiments with the STAC tandem SH3 domains and the C3 peptide revealed clear differences between the STAC2 and STAC3. STAC3 exhibited extensive NMR chemical shift perturbations in both SH3 domains, yet only the first SH3 domain of STAC2 elicited notable CSPs. This suggests that a higher number of amino acid residues of STAC3 is directly involved in the binding of the DHPR II–III loop, which is potentially accounted for by a higher degree of plasticity and a more significant conformational change within the tandem SH3 domains of STAC3 compared to the neuronal isoform (i.e., STAC2 lacks the conformational adaptability of STAC3). The fact that CSPs were only observed in the first SH3 domain of STAC2 (from the direct interaction of STAC2SH3s residues with the II–III loop) is in agreement with the crystal structure of Wong King Yuen et al.^6^ that revealed the binding of a II–III loop peptide fragment to SH3_1. Importantly, when expressed independently of SH3_1, the second SH3 domain of STAC3 exhibited no CSPs upon titration with the C3 peptide (Figure S2), thus indicating no direct interactions. This suggests that binding of the C3 peptide to STAC3SH3s is via SH3_1, which, in turn, elicits an allosteric effect on SH3_2, thereby promoting binding. Alternatively, the II–III loop is anchored to the SH3 domains primarily by SH3_1 and is merely unable to bind to SH3_2 without the presence of the first SH3 domain.

All three STAC isoforms have similar 3D crystal structures of the tandem‐SH3 domains.^6^, ^23^ However, in terms of sequence similarities, the SH3_2 domains are particularly divergent between the three isoforms (Tables S1, S2). Due to the structural similarities between the three STAC proteins,^6^ it is likely that the II–III loop of DHPR binds to the first SH3 domain of all three STAC proteins, but induces a global conformational change in STAC3 alone, due to its unique properties, potentially because of a more distinct amino acid composition of the SH3_2 domain.

With regard to a correlation between the AlphaFold predictions and the corresponding chemical shift perturbations (CSPs) in the NMR experiments, in general, there is a certain amount of consistency in between AlphaFold and the NMR data, for example, the residues displaying the CSPs are clustered in the groove around the W284 residue in the first SH3 domain of STAC3 (Figure 5) where AlphaFold also predicts an interaction with the II–III loop (Figure S3). However, there are also additional residues displaying CSPs located in the second SH3 domain of STAC3 that are not fully consistent with the AlphaFold model. These additional residues most likely reflect the conformation exchange in the STAC3SH3s molecule upon binding of the II–III loop, which is not fully captured in the AlphaFold predictions.

The identified II–III loop‐STAC associations corroborate a wealth of existing studies. Specifically, Polster et al.^5^ indicated that all three STAC proteins promote membrane expression of α_1S_‐subunit of DHPR and co‐localize with the channel, while Wong King Yuen et al.^6^ suggested that each of the three proteins interacts with the II–III loop. The ability of the neuronal STAC isoforms to bind the II–III loop is not surprising, given the structural similarity between the three STAC proteins and evidence that all STAC proteins are able to modulate the activity of Ca_V_1 channels.^14^, ^17^, ^22^, ^28^ What is unique for STAC3 is that a potential subsequent change in the conformation of the tandem SH3 domains upon II–III loop binding may permit activation of RyR1, inducing Ca^2+^ release from the sarcoplasmic reticulum which leads to muscle contraction. The same sequence of events is unnecessary in neurons, and thus, the expression of STAC1 and STAC2 in neurons suggests that these isoforms have no need to transmit a signal from voltage‐gated Ca^2+^ channels through to RyRs, accounting for the potential lack in their conformational plasticity. These isoform‐specific interactions of STAC3SH3s with the II–III loop are in keeping with the fact that STAC3 reconstitutes the ECC significantly better than STAC1 and STAC2.^1^, ^5^, ^15^ However, what remains to be investigated is whether the flexibility of STAC3SH3s is indeed necessary for the molecular mechanism underlying the skeletal muscle ECC.

In summary, we present novel insights into the molecular mechanism underpinning skeletal muscle ECC, by suggesting that the first SH3 domain of STAC3 binds a specific stretch of residues in the DHPR II–III loop, and potentially causes a global conformational change in STAC3, allowing the additional interaction of the second SH3 domain of STAC3 with the II–III loop. This interaction of the tandem SH3 domains of STAC3 with the II–III loop and any potential conformational change is conceivably crucial in the molecular mechanism of skeletal muscle ECC. Future work will be required to study the molecular flexibility of STAC3 in solution in more detail, as well as the nature of any purported interaction with the RyR1.

## 4 MATERIALS AND METHODS

### 4.1 Expression and purification of protein constructs

The genes for human STAC2SH3s and the DHPR II–III loop were purchased from Synbio Technologies (Monmouth Junction, NJ, USA) in a pUC57 plasmid, and subcloned into a pHUE^AmpR^ expression plasmid.^29^ The gene for human STAC3SH3s was ordered from ATUM (Newark, CA, USA) and likewise cloned into the pHUE^AmpR^ expression plasmid (see Figure S1 for the amino acid sequences of these constructs). The pHUE^AmpR^ plasmid‐encoded fusions of each protein construct with His‐Ubiquitin tags for improved solubility, which were overexpressed in *E. coli* BL21 (DE3) cells following induction by 0.4–0.5 mM isopropyl β‐d‐1‐thiogalactopyranoside (IPTG). After a growth period of 20–24 h, the cells were pelleted and resuspended in Buffer A (27 mM NaH_2_PO_4_, 30 mM Na_2_HPO_4_, 500 mM NaCl, 30 mM imidazole, pH 7.0), supplemented with DNase, RNase, and cOmplete™ Mini EDTA‐free Protease Inhibitor Cocktail tablets (Roche, Switzerland). The re‐suspended cells were then lysed using a French press at 1,500 psi (Thermo Fisher Scientific, Waltham, MA, USA), and the resulting lysate was centrifuged for 30 min at 25,000*g* and 4°C. The soluble sample was purified on HisTrap™ HP 5 mL (GE Healthcare, Chicago, IL, USA) nickel‐affinity columns (eluted with increasing concentrations of imidazole [250–325 mM]) using an ÄKTA Pure chromatography platform (GE Healthcare). The eluate was subjected to overnight dialysis in Buffer A with 1 mM dithiothreitol (DTT) in the presence of ubiquitinase UBP41 to cleave off the His‐Ubiquitin tag. The second purification step was then performed on the resulting samples using the same column, to isolate the target protein. The purity of recombinant protein constructs was assessed and confirmed using SDS‐PAGE gel electrophoresis (see Figure S1).

### 4.2 Surface plasmon resonance (SPR) spectroscopy

SPR spectroscopy experiments were conducted at 20°C on a Biacore 8 K platform (GE Healthcare) in HEPES‐buffered saline (10 mM HEPES‐HCl, pH 7.4, 150 mM NaCl) supplemented with 0.05% Tween‐20 and 3 mM EDTA. The full DHPR II–III loop in 50 mM sodium acetate (pH 4.1) was bound to a CM5 sensor chip (GE Healthcare) via amine coupling, and increasing amounts of STAC proteins were applied at 30 μL/min for 120 s. The analysis of the binding curves, including the determination of the binding affinities, was performed using the Biacore Analysis software (GE Healthcare).

### 4.3 Nuclear magnetic resonance (NMR) spectroscopy

Samples for NMR experiments were prepared as outlined above, with minor modifications for enrichment with ^15^N/^13^C isotopes. Briefly, *E. coli* BL21(DE3) cells were grown in M9 minimal media, with ^15^NH_4_Cl and/or [U‐^13^C]D‐glucose (Cambridge Isotope Laboratories, Tewksbury, MA, USA) as the sole sources of nitrogen and/or carbon, respectively, and induced with 0.4–0.7 mM IPTG. Lysis, HisTrap purification, and de‐ubiquitination were performed as described above. Resulting protein samples were dialyzed into 27 mM NaH_2_PO_4_, 30 mM Na_2_HPO_4_, 200 mM KCl, 10% D_2_O, 0.1 mM sodium trimethylsilylpropanesulfonate (DSS), pH 6.5 (for titration of ^15^N‐labeled DHPR II–III loop with STAC3SH3s), or 27 mM NaH_2_PO_4_, 30 mM Na_2_HPO_4_, 150 mM NaCl, 100 mM glutamic acid, 100 mM arginine, 3 mM EDTA, 10% D_2_O, 0.1 mM DSS, 0.1% NaN_3_, pH 6.8.

^15^N,^13^C‐labeled samples of STAC3SH3s, and STAC2SH3s were concentrated to ~0.5 mM, and triple‐resonance experiments [HNCO, HNCA, HN(CA)CO, HN(CO)CA, CBCA(CO)NH, HBHA(CO)NH] were acquired and analyzed for sequence‐specific NMR assignments. For titration of STAC2SH3s, STAC3SH3s, and STAC3 SH3_2 with the C3 peptide, ^15^N‐only labeled samples were concentrated to ~0.2 mM. For these experiments, lyophilized samples of the C3 peptide were dissolved in 27 mM NaH_2_PO_4_, 30 mM Na_2_HPO_4_, 150 mM NaCl, 100 mM glutamic acid, 100 mM arginine, and 3 mM EDTA, pH 6.8.

All NMR spectra were acquired on Bruker Avance III HD 600 MHz or 800 MHz NMR spectrometers at 20°C, except for the titration of the ^15^N‐labeled II–III loop with STAC3SH3s, which was conducted at 5°C. Spectra were acquired and processed using Bruker TopSpin 3.5 software and referenced relative to the NMR peaks of DSS, which was used as an internal standard. In ^1^H‐^15^N HSQC titration experiments, upon recording of reference spectra, the titrant was added in 0.5:1, 1:1. 2:1, and 4:1 ratio and spectra re‐recorded.

The sequence‐specific assignments and subsequent analyses of the collected NMR spectra were performed using the CCPNMR Analysis 2.4.2 software.^30^ In titration experiments, the weighted CSPs for each residue were calculated based on scaling factors 1 and 0.15 for H and N dimensions, respectively^31^:

$$\Delta =\sqrt{\delta_{H}^{2}+0.15\cdot \delta_{N}^{2}}$$

where ∆ is the weighted CSP, $\delta_{N}$ is the CSP in the nitrogen dimension, and $\delta_{H}$ is the CSP in the hydrogen dimension. Significant CSPs were classified as those greater than 0.1 ppm in magnitude.

### 4.4 AlphaFold modeling

The ColabFold implementation^32^ of Alphafold‐multimer^26^ was used to generate models of human STAC2 and STAC3 in complex with the DHPR α_1S_ II–III loop (residues 665–785). Five models of each complex were produced. The quality of the prediction was probed by the overlay of the top‐ranked models with the available crystal structures of STAC2SH3s and STAC3SH3s. The RMSD over 113 Cα atoms of the STAC2SH3s structure (PDB 6B27)^6^ to the top‐ranked AlphaFold model was 1.45 Å (Figure S1). The top‐ranked template‐free AlphaFold model of STAC3SH3s superimposed with the crystal structure of STAC3SH3s P269R (PDB 6UY7)^23^ with RMSD of 1.1 Å over 111 Cα atoms.

## CONFLICT OF INTEREST

The authors declare no potential conflict of interest.

## AUTHOR CONTRIBUTIONS

**Dmitry Shishmarev:** Formal analysis (equal); funding acquisition (equal); investigation (equal); methodology (lead); visualization (lead); writing – original draft (equal). **Emily Rowland:** Formal analysis (equal); investigation (equal); writing – original draft (equal). **Shouvik Aditya:** Conceptualization (supporting); investigation (supporting). **Srinivasan Sundararaj:** Conceptualization (supporting); investigation (supporting). **Aaron J. Oakley:** Methodology (supporting); software (lead). **Angela F. Dulhunty:** Conceptualization (supporting); writing – review and editing (supporting). **Marco G. Casarotto:** Conceptualization (lead); funding acquisition (equal); project administration (lead); writing – review and editing (lead).

## Supporting information


**Appendix S1.** Supporting Information
