# Supplementary material for: Molecular interactions of STAC proteins with skeletal muscle dihydropyridine receptor and excitation‐contraction coupling
Source: Protein Sci. 2022 Apr 20;31(5):e4311. doi: 10.1002/pro.4311 (PMC9019556; doi:10.1002/pro.4311)
Supplement: Supplementary file 1 — Appendix S1. Supporting Information [file PRO-31-e4311-s001.pdf]

SUPPLEMENTARY MATERIAL

A

```
STAC1      -----NTYVA LYKFVPQENE DLEMRPGDII TLLEDSNEDW WKGKIQDRIG   332
STAC2      -----YSYVA LYKFLPQENN DLALQPGDRI MLVDDSNEDW WKGKIGDRVG   339
STAC3      GFQQSHYFVA LYRFKALEKD DLDFPPGEKI TVIDDSNEEW WRGKIGEKVG   294
                  :** **:*   *:: ** : **: *  :::****:* *:*** :::*

STAC1      FFPANFVQRL QQNEKIFRCV RTFIGCKEQG QITLKENQIC VSS--EEEQD   380
STAC2      FFPANFVQRV RPGENVWRCC QPFSGNKEQG YMSLKENQIC VGVGRSKDAD   389
STAC3      FFPPNFIIRV RAGERVHRVT RSFVGNREIG QITLKKDQIV VQK--GDEAG   342
           *** **: *: : .*.: *   : * * :* *  ::**::**  *     .: .

STAC1      GFIRVLSGKK KGLIPLDVLE NI                                 402
STAC2      GFIRVSSGKK RGLVPVDALT EI                                 411
STAC3      GYVKVYTGRK VGLFPTDFLE EI                                 364
           *:::* :*:*  **.* * *  :*
```

B

•676 •696

EAESLTSAQK AKAEERKRRK MSRGLPDKTE EEKSVMAKKL 705

•716 •736

EQKPKGEGIP TTAKLKVDEF ESNVNEVKDP YPSA**DFPGDD** 745

•756 •776

EEDEPEIPVS PRPRPLAELQ LKEKAVPIPE ASSFF 780

Figure S1. Amino-acid sequences of protein constructs used in this study. (**A**) Multiple sequence alignment of tandem SH3 domains of human STAC1, STAC2, and STAC3, with sequence numbers relative to the corresponding full-length STAC proteins. "*" indicates perfect alignment; ":" indicates strong amino acid similarity; "." Indicates weak amino-acid residue similarity. Sequence alignment was performed using Clustal Omega web server.[1] (**B**) Sequence of the DHPR II-III loop, numbered according to the full sequence of the α_{1S}-subunit of DHPR. The C3 peptide used in this study is highlighted in bold.

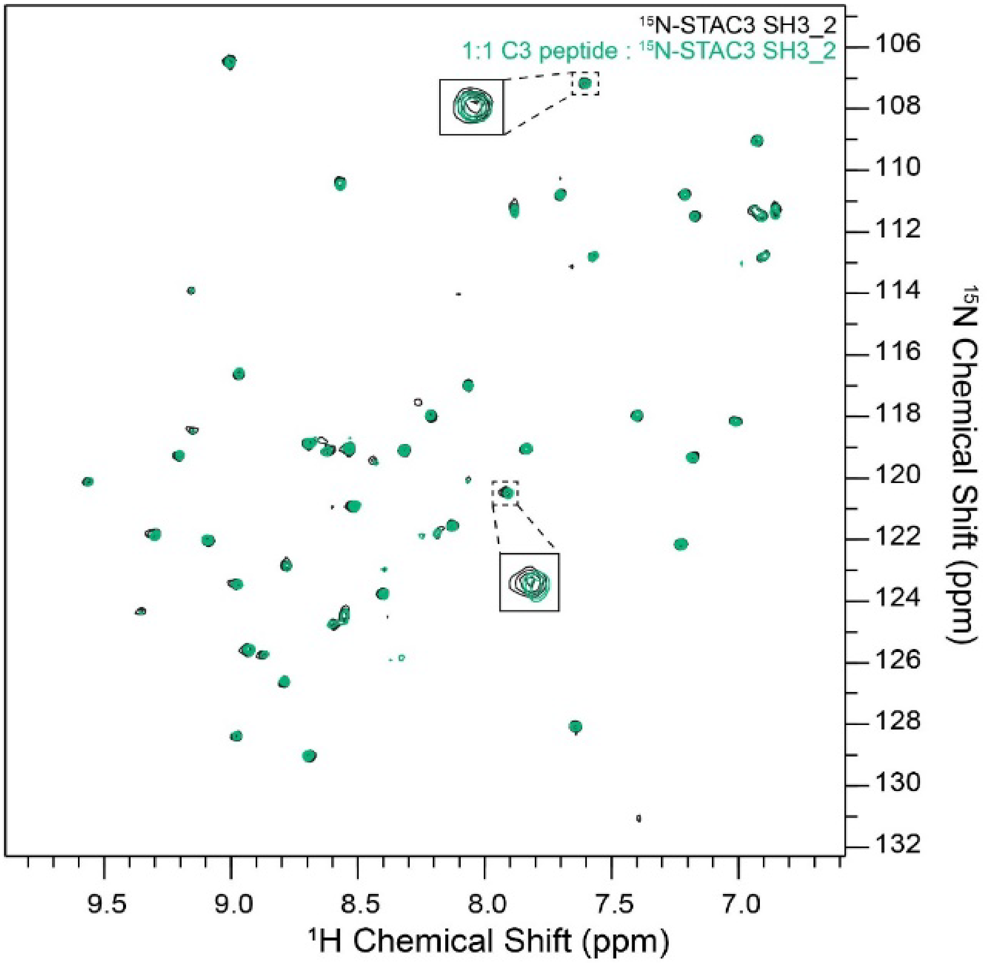

Figure S2. Individual 2nd SH3 domain of STAC3 does not interact with the C3 peptide. ^{1}H-^{15}N HSQC NMR spectra of ^{15}N-labelled STAC3 SH3_2 in the presence (green) and absence (black) of the C3 peptide (1:1 ratio). No substantial difference between the two spectra suggests that the individual STAC3 SH3_2 does not interact with the C3 peptide in solution.

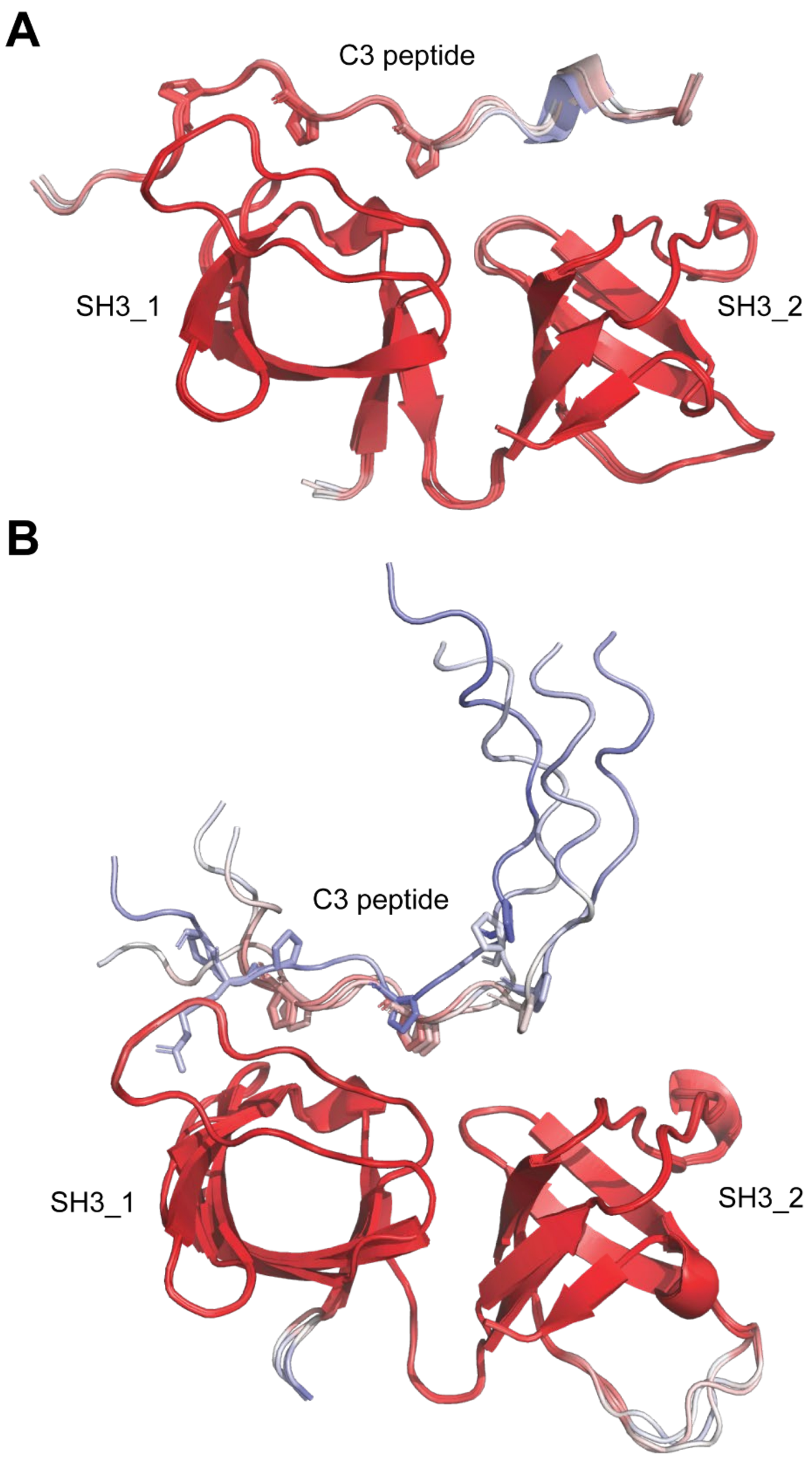

Figure S3. Overlay of the four highest-ranked AlphaFold models of the STAC3SH3s (**A**) and STAC2SH3s (**B**) complexed with the C3 peptide. The cartoons are coloured according to AlphaFold's per-residue confidence score (pLDDT) (blue = low, white = medium, red = high).

Prolines in the C3 peptide residues PEIPLSP (750-756) are shown in stick form in order to highlight the PXXP motifs. For STAC3, the interactions span both SH3 domains, while for STAC2, the interactions are only predicted for the 1st SH3 domain.

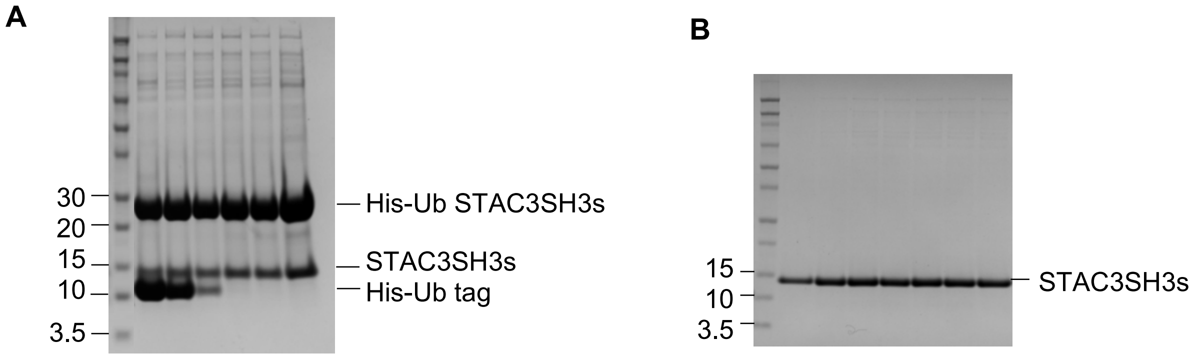

Figure S4. SDS-PAGE gels demonstrating purity of expressed STAC3SH3s samples. (**A**) SDS-PAGE gel obtained after the 1st round of Ni^{2+} affinity purification, exhibiting the presence of His-Ub-tagged STAC3SH3s in samples eluted with 200-325 mM imidazole. The marker to the left of the loaded samples is annotated with sizes of protein bands in kDa. The His-Ub STAC3SH3s fusion construct is ~28 kDa in size. (**B**) SDS-PAGE gel of samples from (**A**) following the cleavage of His-Ub-tag, eluted with 30 mM imidazole. Highly pure STAC3SH3s constructs are observed at the expected size of ~14 kDa.

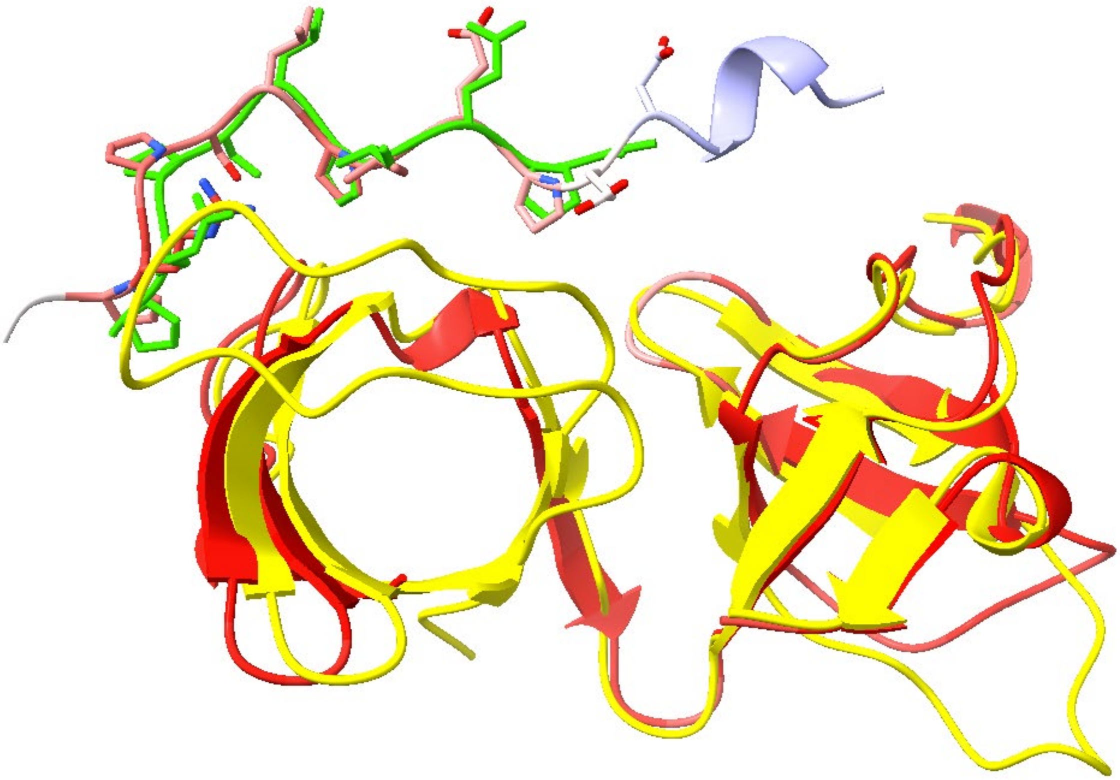

Figure S5. The template-free AlphaFold prediction superimposes closely with the STAC2/II-III loop crystal structure. Overlay of the available X-ray crystal structure of STAC2SH3s (yellow) in complex with the II-III loop peptide (lime) [PDB 6B27][2] and the template-free top-ranked AlphaFold prediction model of STAC3SH3s in the complex with the II-III loop peptide, coloured according to per-residue confidence score (pLDDT) (blue = low, white = medium, red = high). Amino acid residues DEPEIPLSP in the II-III loop (748-756) are shown in stick form in order to highlight the PXXP motifs.

Table S1. Percent identity matrix for SH3_1 domains of STAC proteins[a]

	STAC1	STAC2	STAC3
STAC1	100	71.93	49.12
STAC2	71.93	100	56.14
STAC3	49.12	56.14	100

[a]Created by Clustal2.1[1]

Table S2. Percent identity matrix for SH3_2 domains of STAC proteins[a]

	STAC1	STAC2	STAC3
STAC1	100	57.14	46.43
STAC2	57.14	100	42.86
STAC3	46.43	42.86	100

[a]Created by Clustal2.1[1]

References

1. Madeira F, Park YM, Lee J, Buso N, Gur T, Madhusoodanan N, Basutkar P, Tivey ARN, Potter SC, Finn RD et al. (2019) The EMBL-EBI search and sequence analysis tools APIs in 2019. Nucleic Acids Res 47:W636-W641.
2. Wong King Yuen SM, Campiglio M, Tung CC, Flucher BE, Van Petegem F (2017) Structural insights into binding of STAC proteins to voltage-gated calcium channels. Proc Natl Acad Sci U S A 114:E9520-E9528.
